# Prodrugs of Nonsteroidal Anti-Inflammatory Drugs (NSAIDs), More Than Meets the Eye: A Critical Review

**DOI:** 10.3390/ijms131217244

**Published:** 2012-12-17

**Authors:** Amjad M. Qandil

**Affiliations:** 1 Pharmaceutical Sciences Department, College of Pharmacy, King Saud bin Abdulaziz University for Health Sciences, Riyadh 11426, Saudi Arabia; E-Mail: qandila@ksau-hs.edu.sa or drqandil@just.edu.jo; Tel.: +966-1-801-1111; 2 Department of Medicinal Chemistry and Pharmacognosy, Faculty of Pharmacy, Jordan University of Science and Technology, Irbid 22110, Jordan

**Keywords:** anti-inflammatory, cyclooxygenase, codrug, mutual prodrug, NO-NSAIDs, NSAIDs, Phospho-NSAIDs, prodrug

## Abstract

The design and the synthesis of prodrugs for nonsteroidal anti-inflammatory drugs (NSAIDs) have been given much attention by medicinal chemists, especially in the last decade. As a therapeutic group, NSAIDs are among the most widely used prescribed and over the counter (OTC) medications. The rich literature about potential NSAID prodrugs clearly shows a shift from alkyl, aryalkyl or aryl esters with the sole role of masking the carboxylic acid group, to more elaborate conjugates that contain carefully chosen groups to serve specific purposes, such as enhancement of water solubility and dissolution, nitric oxide release, hydrogen sulfide release, antioxidant activity, anticholinergic and acetylcholinesterase inhibitory (AChEI) activity and site-specific targeting and delivery. This review will focus on NSAID prodrugs that have been designed or were, later, found to possess intrinsic pharmacological activity as an intact chemical entity. Such intrinsic activity might augment the anti-inflammatory activity of the NSAID, reduce its side effects or transform the potential therapeutic use from classical anti-inflammatory action to something else. Reports discussed in this review will be those of NO-NSAIDs, anticholinergic and AChEI-NSAIDs, Phospho-NSAIDs and some miscellaneous agents. In most cases, this review will cover literature dealing with these NSAID prodrugs from the year 2006 and later. Older literature will be used when necessary, e.g., to explain the chemical and biological mechanisms of action.

## 1. Introduction

### 1.1. Prodrugs

**Figure 1:**
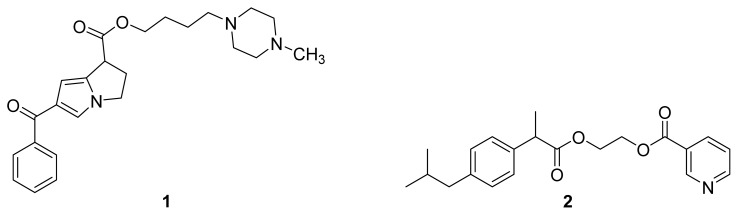
The chemical structures of a ketorolac prodrug (**1**) and a codrug of ibuprofen and nicotinic acid (**2**).

The terms prodrug or proagent were introduced by Adrien Albert in 1958 and started to gain popularity by the 1960s [1,2]. Prodrugs can be defined as “bioreversible derivatives of drug molecules that undergo an enzymatic and/or chemical transformation *in vivo* to release the active parent drug, which can then exert the desired pharmacological effect” [3]. In other words, the desired pharmacological action is expected to be that of the parent drug, whereas the prodrug should have no or negligible biological activity. In the most common cases, a prodrug is synthesized from a parent drug by covalently linking it, with or without a carrier, to a pharmacologically inert promoiety, which can be cleaved enzymatically and/or chemically upon administration, releasing the parent drug [4,5]. When the promoiety is not pharmacologically inert, *i.e.*, another drug molecule, a codrug (mutual prodrug) is formed [6]. In a codrug, the pharmacological activity will be exerted when the codrug is enzymatically and/or chemically cleaved to form the two parent drugs. Examples of a prodrug of ketorolac (**1**) [7] and a codrug of ibuprofen and nicotinic acid (**2**) [8] are shown in Figure 1.

The goals of prodrug design include but are not limited to the improvement of physicochemical properties [9,10], enhancement of biopharmaceutical profile [10,11], reduction of side effects [9,11], obtaining additive or synergistic effects [6,12], targeted delivery [11,13] or optimization for the route of administration [7,14,15].

### 1.2. Non-Steroidal Anti-Inflammatory Drugs (NSAIDs)

**Figure 2:**
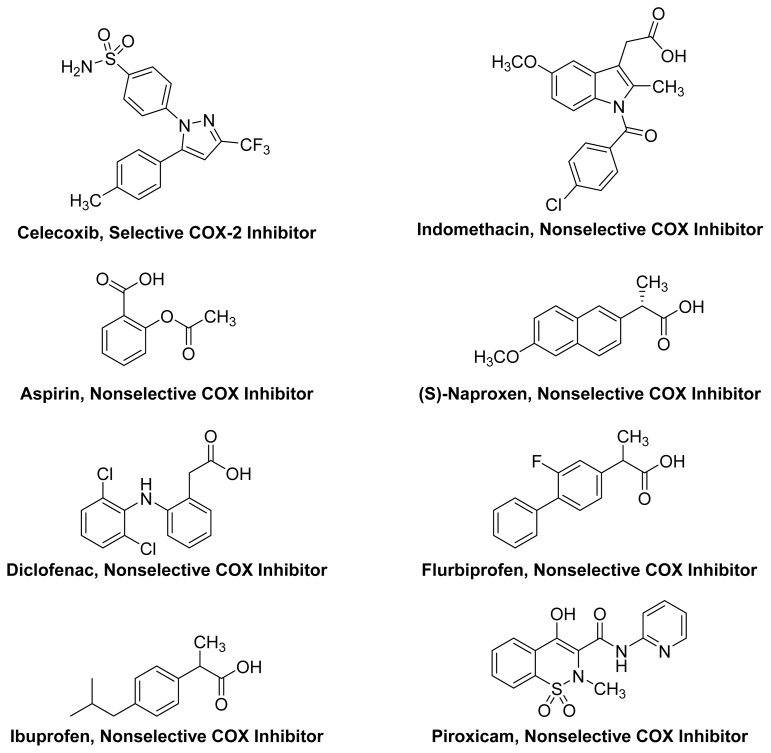
Chemical structures of some commercially available NSAIDs.

Non-Steroidal Anti-Inflammatory Drugs (NSAIDs) are a diverse group of compounds that are mainly used to reduce fever, pain and inflammation. NSAIDs are among the most frequently used classes of medications, which, as of 2009, represented a market worth more than $9 billion among prescribed medications in the USA [16]. NSAIDs exert their pharmacological action by inhibiting the synthesis of prostaglandins (PGs) by non-selectively blocking *cyclooxygenases 1* and *2* (COX-1 and COX-2) or by selectively blocking COX-2. Inhibition of COX-1 is also responsible, in part, for gastrointestinal side effects, which are the most frequent side effects of NSAIDs [17]. Non-selective COX inhibitors have other contributors to their gastrointestinal side effects, which are the carboxylic acid group in compounds, such as aspirin, ibuprofen and diclofenac, and the acidic enolic group in oxicams, such as piroxicam [18]. These acidic groups cause local irritation upon oral administration, which can lead to the clinically observed gastrointestinal side effects either independently or in tandem with inhibition of the COX-1 enzyme. In a recent report, Lanas *et al.* (2011) have concluded that more than 90% of the treated patients with osteoarthritis are at increased GI risk, with 60% of them at high risk [19]. Selective COX-2 inhibitors, such as celecoxib, have been designed, synthesized and clinically introduced as gastrointestinal (GI)-sparing NSAIDs. These compounds are not devoid of side effects, as they can cause adverse cardiovascular events [20,21]. The chemical structures of some commercially available NSAIDs are shown in Figure 2.

### 1.3. NSAID Prodrugs

**Figure 3:**
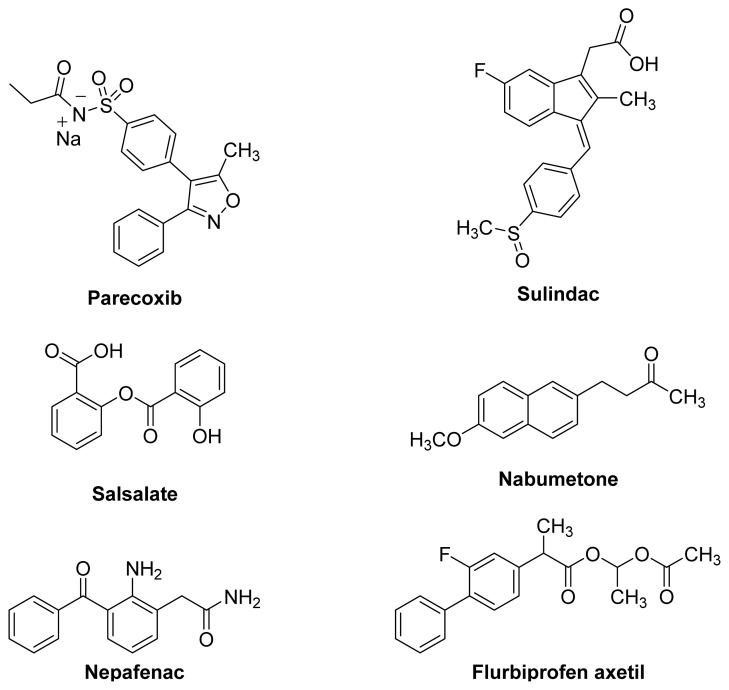
The chemical structures of some commercially available NSAID prodrugs.

In addition to the large number of chemical and pharmacological research reports on NSAID prodrugs, they have been discussed in many collective reviews [3,22–27] and a few specialized reviews [28]. After examining this mounting literature on NSAID prodrugs, it becomes obvious that most of the efforts to design prodrugs of non-selective COX inhibitors were devoted to masking the free acidic groups in these molecules in order to protect the gastrointestinal tract (GIT) from local irritation. By contrast, COX-2 inhibitors were converted into prodrugs mainly to obtain derivatives with enhanced water solubility for parenteral use or to improve oral bioavailability [29–31]. Despite extensive research in designing, synthesizing and evaluating potentially useful NSAID prodrugs, only a few examples (Figure 3) have made it into clinical use, and some of them may have not been the result of rational drug design.

A careful look at the state of science in NSAID prodrugs reveals that there is an emerging group of NSAID prodrugs that possesses pharmacological activity of its own without the need to liberate the parent NSAID. From here on, this action will be referred to as intrinsic pharmacological activity. This group of NSAID prodrugs with intrinsic pharmacological activity, in my opinion, has not been given due attention, nor has it been given a proper designation. This review will examine recent literature, mainly since 2006, but with some older relevant reports related to NSAID prodrugs and codrugs that were designed or then later found to possess intrinsic pharmacological activity. Such activity can contribute to their potential therapeutic value, enhance their safety profile or introduce potentially new biological activity.

Most often, when prodrugs are designed, little attention is given to the possibility of intrinsic pharmacological activity for the newly synthesized chemical entities. That is most likely due to the fact that it is not practical to test the new molecule for all of the possible “expected and unexpected” pharmacological activities. A virtual *in silico* screening of the designed molecules might help in this matter. A second reason is the notion that prodrugs, especially esters, are expected to be short-lived *in vivo*, and hence, there would be no point in testing them for possible pharmacological activity *in vitro*. The question then is, why does the intrinsic pharmacological activity of a prodrug molecule matter? Prodrugs, like most other drugs, are usually taken orally, and in most instances, their stability in simulated GIT conditions is tested. Acceptable chemical stability in the GIT is desired or even required for prodrugs, especially if they are NSAID prodrugs. Taking that into consideration, one nevertheless cannot ignore the pharmacological effect of prodrug molecules that have intrinsic activity. Furthermore, particularly for prodrugs that have relatively long plasma half-lives, the possibility of a systemic action of the prodrug molecule must be considered. These should not be regarded as negative implications; often they can be quite the opposite. As will be discussed later, there are many examples of prodrugs that have intrinsic pharmacological activities that can potentially enhance or change the effect of the parent drug and/or alleviate its side effect(s).

The main groups of NSAID prodrugs that will be discussed in this review are the nitric oxide-releasing NSAIDs (NO-NSAIDs), NSAID prodrugs with anticholinergic or acetylcholinesterase inhibitory (AChEI) activity, phospho-NSAIDs and two agents of special interest.

## 2. NO-NSAIDs

### 2.1. Introduction

Cooperatively, nitric oxide (NO) and prostaglandins (PGs) play a role in the maintenance of the gastrointestinal mucosa, which means that the reduction in the levels of one will be compensated by an increase in the levels of the other [32]. So, it is not surprising that there are inducible enzymes in their biosynthetic pathways, namely *inducible nitric oxide synthase* (iNOS) and COX-2 [33,34]. A plethora of synthetic and pharmacological reports of nitric oxide-releasing prodrugs of NSAIDs (NO-NSAIDs) has emerged with the rationale that NO will compensate for the reduced protective effects of PG’s caused by the inhibition of their synthesis by NSAIDs. Although selective COX-2 inhibitors are GI-sparing, the withdrawal of rofecoxib and valdecoxib from the market in 2004 due to cardiovascular complications [35] might have increased the momentum to find GI-sparing NSAIDs that are not selective COX-2 inhibitors. Furthermore, the popularity of NO-NSAID in drug discovery efforts has been increased due to the fact that NO releasing agents, like nitroglycerin, have a well-known vasodilatory effect that can counteract the increase in blood pressure caused by NSAIDs [36].

**Figure 4:**
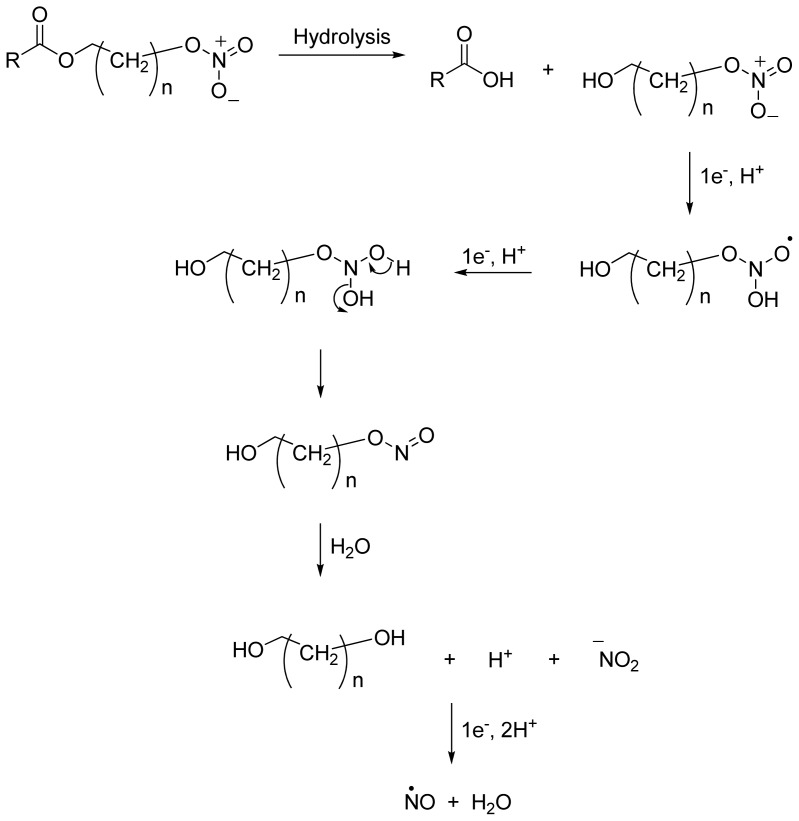
Chemical structure and hydrolysis of simple NO-NSAIDs.

NO-NSAIDs can be defined as codrugs if nitric oxide is considered to be a drug molecule that has its own pharmacological activity, and sometimes, they are given the rather vague designation of hybrid prodrugs. Examples of NO-NSAIDs are numerous in the literature and were summarized in an informative review by Koc and Kucukguzel [28]. Simple NO-NSAIDs consist of an NSAID molecule connected via an alkyl spacer to a nitrate group (-ONO_2_), as seen in Figure 4, which also illustrates the mechanism of NO release. *In vivo*, a typical NO-NSAID is initially hydrolyzed into the parent NSAID and a nitrate-containing alcohol. A two-electron reduction cascade of the nitrate group followed by dehydration leads to the formation of a nitrite derivative. Hydrolysis of this nitrite derivative liberates a diol and a nitrite anion. Finally, the nitrite anion is further reduced to afford a nitric oxide molecule [37]. It is believed that the *CYP3A4-NADPH-cytochrome P450 reductase* system is involved in nitric oxide formation from organic nitrates [38]. In addition to nitrates, other NO-releasing groups have been incorporated in NO-NSAIDs [39–47], a summary of which can be found in Table 1.

**Figure 5:**
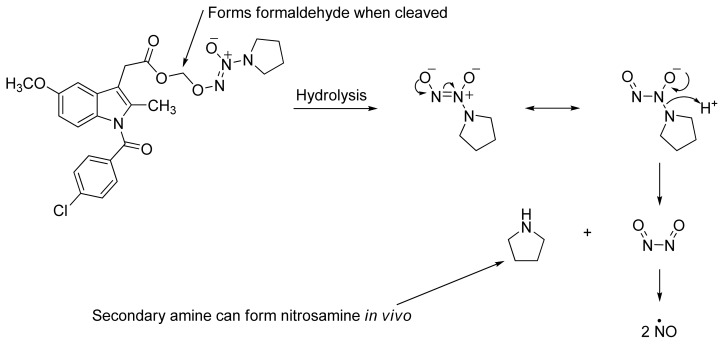
*In vivo* release of nitric oxide from NONOate-containing NO-NSAIDs.

Among the groups in Table 1, the NONOate (*N*-diazen-1-ium-1,2-diolate) derivatives are considered promising NO-donors that hold some advantages over organic nitrates. The NONOates spontaneously release NO in physiological media (Figure 5), whereas the organic nitrates need metabolic reduction to release NO [48]. In addition, the NONOate group can be derivatized, which opens the possibility of designing derivatives for targeted drug release. Finally, NONOates release higher equivalents of NO per prodrug molecule than organic nitrates [49]. The mechanism of NO release from NONOates is shown in Figure 5. It can be seen that after initial hydrolysis, the resultant NONOate is stabilized by resonance [50]. The mechanism also reveals the main disadvantages of NONOates, which are the formation of formaldehyde and a secondary amine that can potentially form toxic metabolites, such as nitrosamines [43,45].

### 2.2. NO-NSAIDs with Intrinsic Pharmacological Activity

With regard to NO-NSAIDs, the gastroprotective effect of these prodrugs is assumed to be due to the release of NO *in vivo*. This assumption may be questioned after considering a comparison conducted by Chattopadhyay *et al.* (2010) between the *in vivo* safety profiles of nitrate and NONOate-containing NSAID prodrugs [48]. The researchers found that there were no significant differences between the safety profiles of both groups of NO-NSAIDS, which led to the conclusion that the amount of NO released per prodrug molecule may not be the only determining factor in the gastroprotective effect of these prodrugs [48].

**Figure 6:**
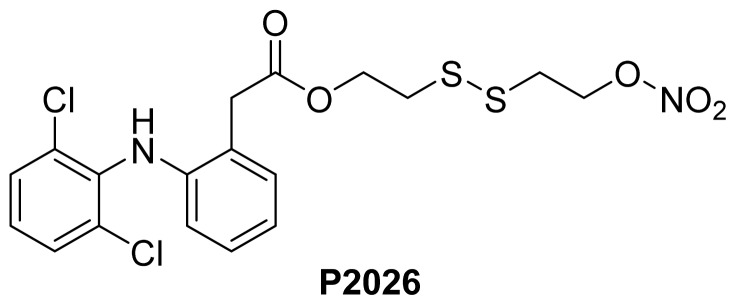
The chemical structure of **P2026**.

Before discussing NO-NSAIDs with intrinsic pharmacological activity, it should be mentioned that there are reports of NO-NSAIDs that clearly show the quantitative release of the parent NSAID and nitric oxide *in vivo*, *i.e.*, fulfilling the definition of true prodrugs. For example, **P2026** (Figure 6), is a nitrate-containing prodrug of diclofenac with a typical plasma release profile for the parent NSAID *in vivo.* There were no detectable levels of the intact prodrug molecule after 2 min, and it released nitric oxide in a predictable dose-dependent manner [51]. Therapeutically, this prodrug showed an anti-inflammatory profile similar to that of diclofenac and produced no visible or microscopic gastric lesions.

**Figure 7:**
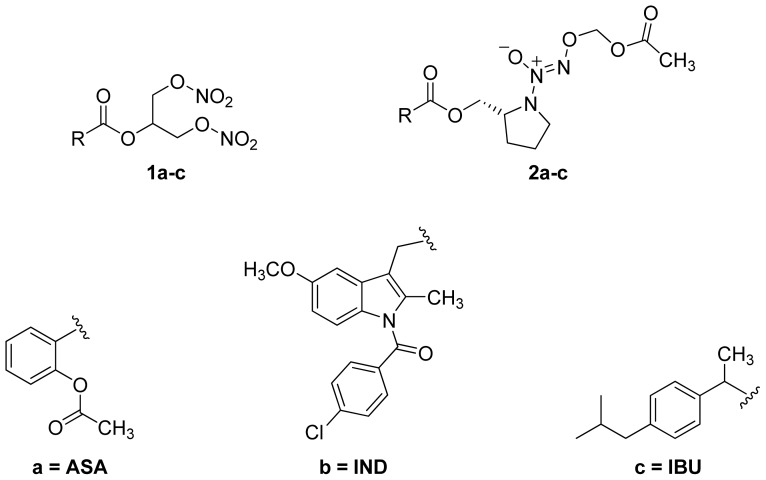
The chemical structures of the glyceryl dinitrate esters **1a**–**c** and NONOate-containing esters **2a**–**c** of aspirin (**a** = **ASA**), indomethacin (**b** = **IND**) and ibuprofen (**c** = **IBU**).

**Figure 8:**
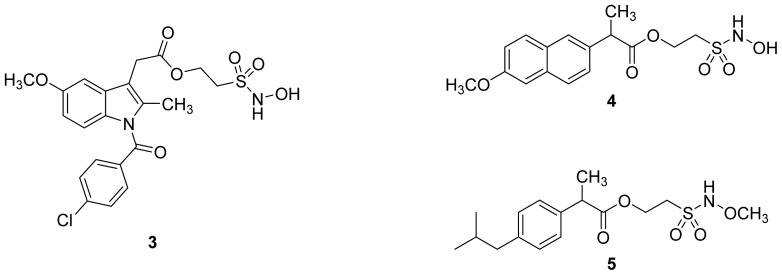
Chemical structures of the ethanesulfohydroxamic acid esters of indomethacin (**3**) and naproxen (**4**) and the methyl ether of ibuprofen ethansulfonhydroxamic acid ester (**5**).

**Figure 9:**
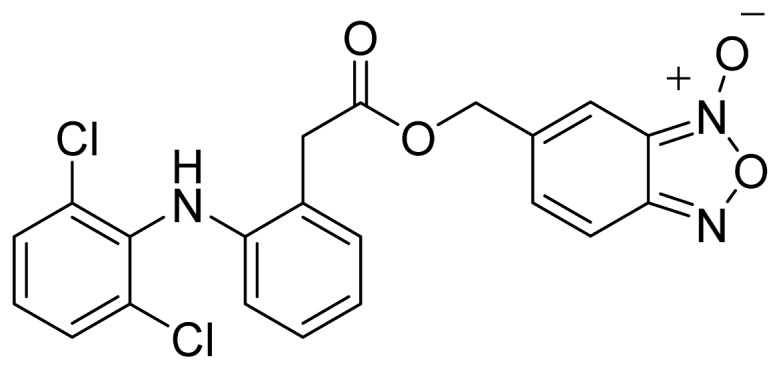
The chemical structure of a NO-releasing ester of diclofenac (**6**).

**Figure 10:**
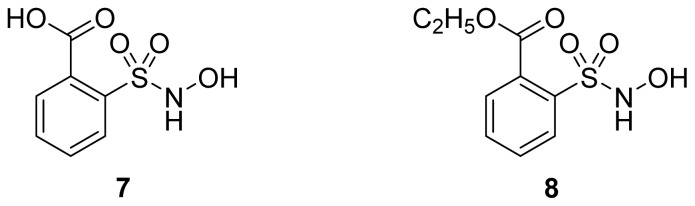
The chemical structures of 2-hydroxysulfamoylbenzoic acid (**7**) and ethyl 2-hydroxysulfamoylbenzoate (**8**).

**Figure 11:**
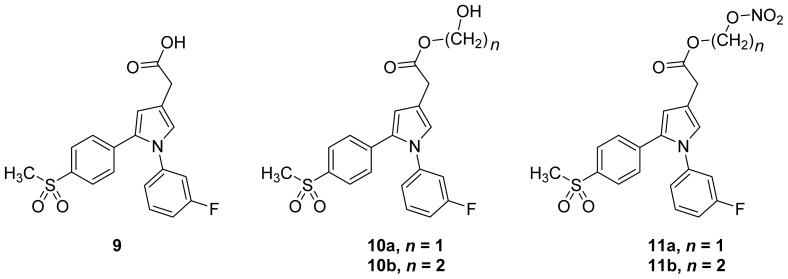
The chemical structures of diarylpyrrole acetic acid **9**, the hydroxyethyl and hydroxypropyl ester intermediates (**10a** and **10b**) and their corresponding nitrate esters (**11a** and **11b**).

**Figure 12:**
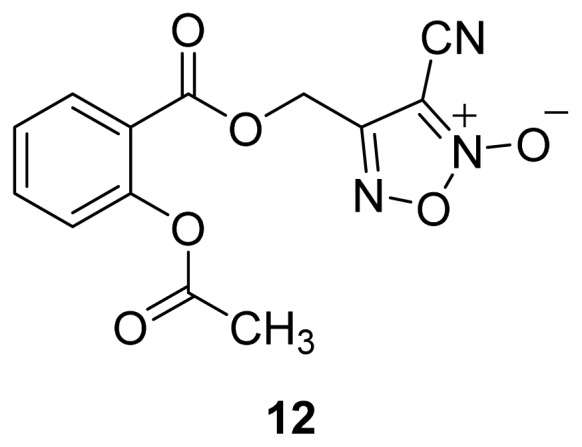
The chemical structure of the furoxan containing ester of aspirin **12**.

By contrast, many reports of potentially useful NO-NSAIDs do not provide such a detailed profile. In addition, some recent reports include *in vitro* molecular details and COX inhibition assays, which suggest that NO-NSAIDs might have intrinsic pharmacological activity that was not intended in the original design of the prodrug. The following paragraphs discuss some of the most interesting and well-documented examples of such compounds:

1. A group of glyceryl dinitrate esters (**1a**–**c**) and NONOate-containing prodrugs (**2a**–**c**) of aspirin (**a** = **ASA**), indomethacin (**b** = **IND**) and ibuprofen (**c** = **IBU**) (Figure 7) have been synthesized and evaluated *in vivo* and *in vitro* for their anti-inflammatory activity [52]. Although the ibuprofen and indomethacin esters showed *in vivo* activity comparable to their parent NSAIDs, the aspirin esters showed less than half the activity of aspirin. Furthermore, none of them showed *in vitro* inhibitory activity against COX-1; rather, they all showed inhibitory activity against COX-2 (*in vitro* IC_50_ = 0.6–9.3 μM) that was comparable to or greater than that of their parent NSAIDs. These results pose two questions: was the *in vivo* biological activity of these compounds due solely to the parent NSAIDs? And, second, was the GI-sparing profile only the result of NO release?
2. The ethanesulfohydroxamic acid esters of indomethacin (**3**) and naproxen (**4**) were synthesized and evaluated as NO-NSAIDs [47] (Figure 8). The indomethacin ester **3** was a selective COX-2 inhibitor (IC_50_ = 0.42 μM) *in vitro*, with an *in vivo* ID_50_ of 19.1 μmol/kg compared to 11.7 μmol/kg for indomethacin. Furthermore, the non-NO-releasing hydroxamic acid ester of ibuprofen **5** showed comparable *in vivo* potency (78.9% inhibition of inflammation at 327 μmol/kg oral dose) to its NO-releasing hydroxamic acid (79.5% inhibition of inflammation at 327 μmol/kg oral dose). It is worth mentioning that the IC_50_ of **5** against COX-2 was 0.63 μM. This adds more skepticism to the accepted notion(s) that NO-releasing esters of NSAIDs are mere prodrugs and/or that NO release is essential for their GI-sparing profile. The possibility that compound **5** is a prodrug still exists, but no investigation of this possibility was performed.
3. The ester of diclofenac (**6**) (Figure 9) contains benzofuroxan as the NO-releasing group. Ester **6** was synthesized and evaluated *in vitro* and *in vivo* for the reduction of PGE_2_ and TXB_2_ levels in plasma. This ester was found to be more effective in inhibiting PGE_2_ synthesis than TXB_2_ synthesis *in vitro*. This finding suggests that this NO-diclofenac ester is more selective for COX-2 [46].
4. 2-Hydroxysulfamoylbenzoic acid **7** and its ethyl benzoate ester **8** (Figure 10), which are nitric oxide-releasing analogs of aspirin, were reported by Kaur *et al.* (2012) [53]. Although not prodrugs, these compounds show that the simple backbone of aspirin, a nonselective COX inhibitor, can be modified to become a selective COX-2 inhibitor [53]. The pharmacological evaluation of compounds **7** and **8** revealed that hydroxamic acid **8** is as a potent and a much more selective COX-2 inhibitor than celecoxib (IC_50_ = 0.09 μM, with more than 1000-fold selectivity). In addition, hydroxamic acid **8** was found to be a 5-lipoxygenase (5-LOX) inhibitor (IC_50_ = 0.4 μM). 5-LOX is an essential enzyme in the biosynthetic pathway of leukotrienes from arachidonic acid. Leukotrienes have an important role in the inflammatory process, and hence, inhibitors of 5-LOX exert an anti-inflammatory action [54]. By contrast, hydroxamic acid **7** was 10 times less potent than both celecoxib and hydroxamic acid **8**, with a COX-2 selectivity comparable to that of celecoxib. Both hydroxamic acid derivatives were effective anti-inflammatory agents *in vivo*, with ED_50_ values of 23.1 μM and 24.5 μM for compounds **7** and **8**, respectively, compared to 10.8 μM for celecoxib and 128 μM for aspirin. It is worth mentioning that these compounds also exhibited a time-dependent release of nitric oxide, which leads to a GIT-safe profile.
5. Biava *et al.* (2012) reported a novel class of diarylpyrrole acetic acid derivatives, which possess the typical scaffold of classic selective COX-2 inhibitors (Figure 11). The researchers showed that **9**, its hydroxyethyl and hydroxypropyl ester intermediates (**10a** and **10b**), and their corresponding nitrate esters (**11a** and **11b**) were all potent selective COX-2 inhibitors *in vitro* (IC_50_ = 0.019-0.083 μM) and considerably more selective than celecoxib. None of the esters (**10a**, **10b**, **11a**, and **11b**) were claimed by the authors to be prodrugs of **9**. Furthermore, molecular docking experiments have shown that the alcohol group in **10a** and **10b** and one of the oxygen atoms of the nitrates in **11a** and **11b** form one or more hydrogen bonds with amino acid side chains in the active site of COX-2. Furthermore, the nitrate ester **11a** exhibited an *in vitro* vasorelaxing effect comparable to that of nitroglycerin [55]. This vasorelaxing effect can be considered the rationale behind designing NO-Coxibs.
6. Finally, an example that previously has gone unnoticed is the furoxan-containing ester of aspirin **12** (Figure 12), which possesses pronounced anti-inflammatory and anti-platelet action *in vivo* and very low gastric side effects without any detectable hydrolysis to aspirin [42].

### 2.3. The Role of the Linker in NO-NSAIDs

**Figure 13:**
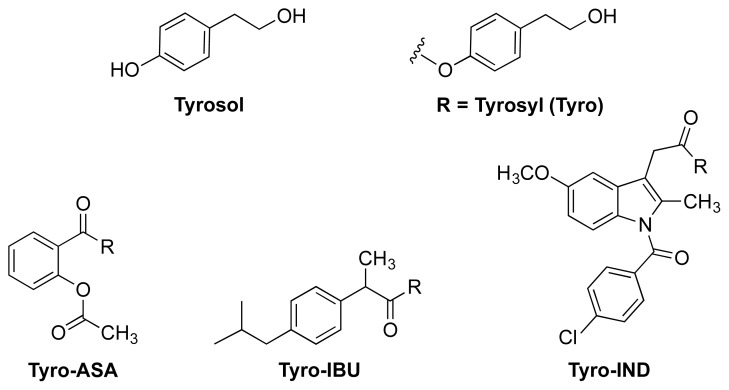
The chemical structures of tyrosol and tyrosyl-NSAID prodrugs **Tyro-IBU**, **Tyro-IBU** and **Tyro-IND**.

In the synthesis of NO-NSAIDs, a linker (spacer) is required in order to attach the NO-releasing group covalently to the carboxylic acid group in the NSAID molecule. Ideally, the linkers are pharmacologically inert and are chosen based on their hydrolysis kinetics. In very few cases, these linkers can impart pharmacodynamics that affect the mechanism of action of prodrugs. Two interesting examples can be of help to illustrate this point and are relevant to the current discussion. The first example is the use of tyrosol (Figure 13) as a potential linker. The recently reported tyrosolyl (Tyro) esters of aspirin (**Tyro-ASA**) and ibuprofen (**Tyro-IBU**) have been synthesized as intermediates for a proposed synthesis of NONOate-containing prodrugs of the two respective NSAIDs. These tyrosolyl intermediates were found to be effective as anti-inflammatory agents (*in vivo* potency similar to the parent drugs) with a 13- to 27-fold reduction in ulcerogenicity index (UI), and hence, they were not converted to the corresponding NO-NSAIDs [49]. To test the concept further, the authors also synthesized the indomethacin analog (**Tyro-IND**) (Figure 13). The three esters were found to be active *in vivo* and *in vitro*. Not only that, but **Tyro-IBU** and **Tyro-IND** exhibited high COX-2 selectivity, reaching more than 30,000-fold in case of **Tyro-IBU** (*in vitro* IC_50_ against COX-2 = 0.01 nM). Although *in vivo* metabolism leading to liberation of the parent NSAID is a possibility, the above results open the door to speculation about the role of the selective COX-2 inhibitory activity of **Tyro-IBU** and **Tyro-IND** in systemic GIT protection. In addition, esterification masks the irritant-free carboxylic acid groups of the parent NSAID and that will help to prevent local injury.

**Figure 14:**
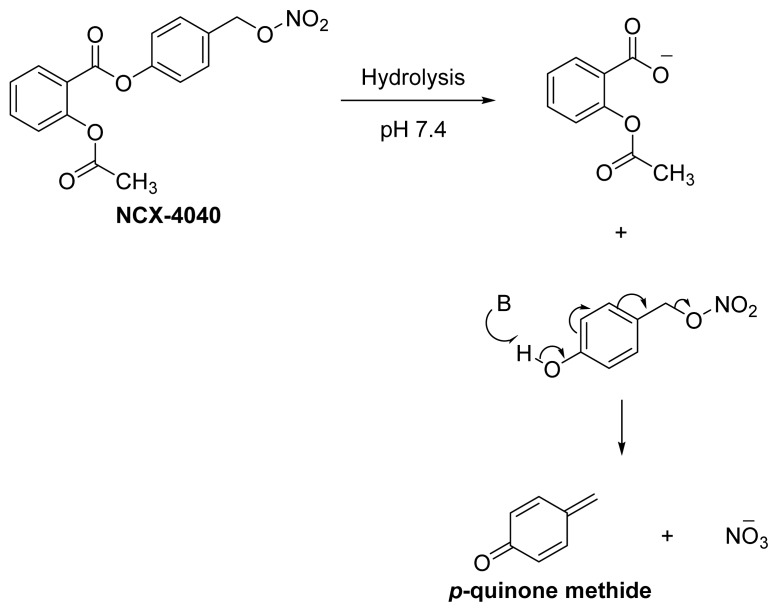
The chemical structure of **NCX-4040** and the mechanism of the formation of *p*-quinone methide.

The second example is the use of p-hydroxybenzylalcohol as a linker in **NCX-4040** (Figure 14). **NCX-4040**, an NO-ASA, has been shown to possess anticancer activity in numerous reports [56–60]. It has been proposed that the activity of **NCX-4040** is mediated by the electrophilic *p*-quinone methide species, which is formed via a 1,4-elimination reaction of the nitrate ester of 4-hydroxybenzylalcohol in which the nitrate plays the role of the leaving group [61,62]. It can be seen that neither aspirin’s own intrinsic activity nor the nitrate’s NO-releasing potential play any role in the pharmacological activity of **NCX-4040**. The overall pharmacological activity of **NCX-4040** is dictated by the unforeseen role that was played by “the linker”.

### 2.4. NO-NSAIDs and *in Vivo* Hydrolysis

**Figure 15:**
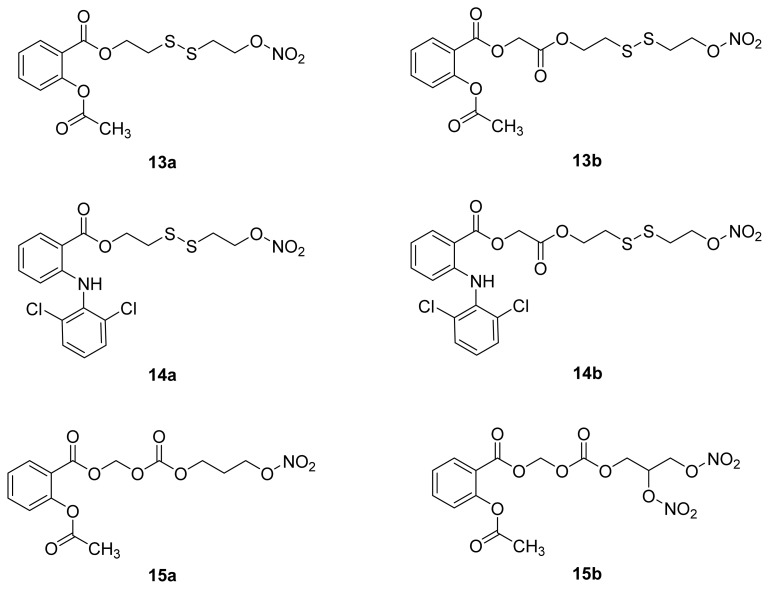
The chemical structures of NO-aspirins and NO-diclofenac prodrugs **13a**, **13b**, **14a**, **14b**, **15a** and **15b**.

As mentioned in Section 2.2, immediate hydrolysis or a very short *in vivo* plasma half-life of the ester molecule can indicate a minor or no role for the intact prodrug in the observed *in vivo* pharmacological activity. The NO-Aspirins (**13a** and **13b**) and NO-Diclofenacs (**14a** and **14b**) (Figure 15) were shown to hydrolyze immediately upon absorption, as none of the intact esters were detected in plasma [63]. This observation negates the possibility that the intact esters participate in the observed *in vivo* activity. Fast plasma hydrolysis also has been reported for the prodrugs **15a** and **15b**[64] (Figure 15).

**Figure 16:**
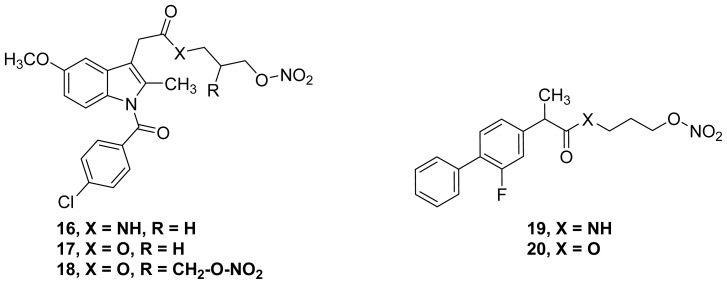
The chemical structures of nitrate-containing amide of indomethacin **16** and esters **17** and **18** and the nitrate-containing amide of flurbiprofen **19**, and ester **20**.

Unfortunately, most of the literature dealing with “prodrugs” relevant to this current review lacks plasma or *in vivo* half-life data. Actually, whether to classify an *in vitro* active NO-NSAID prodrug, a new intrinsically active drug or a drug with mixed action depends on the extent to which the parent drug is released *in vivo* or, at least, in an appropriate *in vitro* hydrolysis model. Two examples can be used to illustrate this point. The first example is the nitrate-containing amide of indomethacin **16** (Figure 16), which is not a prodrug, because its stable amide linkage is not hydrolyzed for a timely release of the parent compound. This compound was shown to be a nitric oxide-releasing, mildly selective COX-2 inhibitor and an effective *in vivo* anti-inflammatory agent [65]. By contrast, its ester analogs **17** and **18** (Figure 16), are considered to be NO-releasing prodrugs of indomethacin, due to the observed fast hydrolysis of the ester linkages in these molecules [66,67]. An additional example is the nitrate-containing amide of flurbiprofen **19** that was synthesized and tested as a metabolically more stable analog of **20**; both compounds are effective inhibitors of amyloidogenesis [68].

To conclude, many of the NO-NSAIDs that have been recently synthesized and pharmacologically tested were proven to possess intrinsic pharmacological activity at least *in vitro* and in many cases *in vivo*. Due to the absence of metabolic and chemical stability data in most of these reports, one cannot decide whether or not this intrinsic pharmacological activity contributes to the overall *in vivo* pharmacological activity observed by those NO-NSAIDs.

## 3. Anticholinergic NSAIDS and AChEI-NSAIDs

### 3.1. Introduction

Anticholinergic agents decrease gastric acid secretion by blocking M_1_ muscarinic receptors [69] and reduce GIT motility by blocking M_2_ and M_3_ muscarinic receptors [70]. These effects lead to optimal blood flow and increased oxygen supply, which can protect against ulcers and promote rapid ulcer healing [71,72]. These effects were the driving force behind the design and synthesis of NSAID prodrugs with non-selective anticholinergic action. These prodrugs were intentionally designed to possess local intrinsic anticholinergic pharmacological activity in the GIT before absorption [71,73,74]. By contrast, NSAID prodrugs with acetylcholinesterase inhibitory activity (AChEI-NSAIDs) are intentionally designed as drugs that can alleviate the inflammatory effect of blistering agents, such as sulfur mustard (2,2′-dichloroethyl sulfide, SM) [75,76]. The cholinergic anti-inflammatory pathway involves parasympathetic deactivation of macrophages via an acetylcholine receptor. It has been shown that stimulation of the vagus nerve suppresses inflammation [77]. AChEIs lead to an increase in the levels of available acetylcholine for receptor binding and are recognized to exert anti-inflammatory action.

### 3.2. Anticholinergic NSAIDs

**Figure 17:**
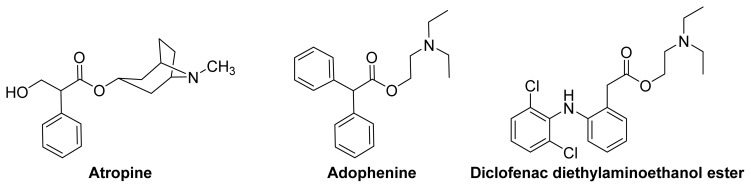
The chemical structures of two anticholinergic compounds, atropine and adophenine and diclofenac diethylaminoethanol ester.

When NSAIDs are converted to *N*,*N-*disubstituted aminoethyl esters, the new compounds fit within the classical pharmacophore of anticholinergic agents (Figure 17).

**Figure 18:**
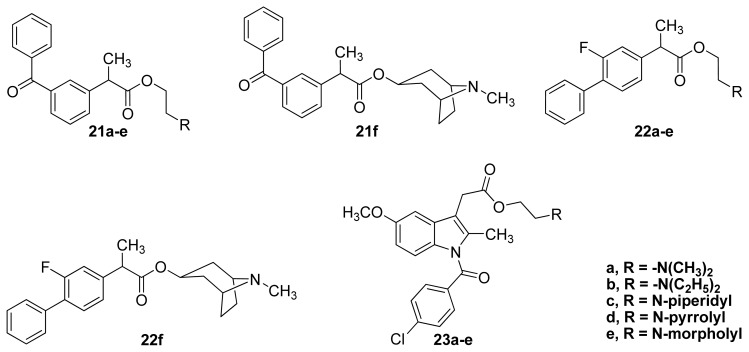
The chemical structures of the aminoethyl esters of ketoprofen **21a**–**f**, flurbiprofen **22a**–**22f** and indomethacin **23a**–**e**.

There have been a few examples of these NSAID prodrugs in the literature [71,73,74]. In this regard, aminoethyl esters of ketoprofen **21a**–**f**, flurbiprofen **22a**–**22f** and indomethacin **23a**–**e** incorporating open and cyclic amines were synthesized and evaluated (Figure 18 and Table 2).

Most of the esters released 17.9%–79.9% of the parent NSAID in human plasma after 2 h and were demonstrated to be competitive reversible antagonists with pA_2_ values ≤7.58 compared to 8.20 for atropine sulfate [71,73,74]. pA_2_ reflects the affinity of atropine for its receptor and is defined as “the negative logarithm of the molar concentration of an equilibrium competitive antagonist, which reduces the effect of a double concentration of agonist to that of a single one” [78]. In addition, these esters exhibited anti-inflammatory action comparable to their parent NSAIDs, but with significant reduction in the ulcerogenic index (UI). Edema volume was measured using the carrageenan-induced rat paw edema model. It is unclear whether this decrease in UI is due to their anticholinergic action and/or the masking of the carboxylic acid group. These esters are expected to be stable in the GIT (*t*_1/2_ at pH 7.4 = 34–505 h), and hence, they can exert a local GIT anticholinergic effect and, at the same time, keep the carboxylic acid group from locally irritating the stomach. In all the above examples, the intact prodrug molecules will be responsible for the anticholinergic activity, then, after hydrolysis, the parent NSAIDs will exert their anti-inflammatory activity.

The data demonstrate that some of the esters were very stable in human plasma, indicating that an ester linkage is not always prone to fast enzymatic hydrolysis. In those cases, the intrinsic activity of the intact ester might play a role in the observed *in vivo* profile of the prodrug. That is, it is no more obvious than in the surprising case of ketoprofen ester **21f**, which possessed *in vivo* anti-inflammatory activity without detectable hydrolysis in human plasma after 2 h. This *in vivo* activity must be due either to an intrinsic anti-inflammatory activity of the intact molecule or to hydrolysis of the ester by different pathways, e.g., liver hydrolytic enzymes. Actually, Halen *et al.* hinted at such a possibility, as it was reported earlier that the indomethacin ester **23c** processes selective COX-2 inhibitory activity [79]. The dilemma might be more difficult to resolve, especially because **22f**, flurbiprofen’s analog of **21f**, which showed no hydrolysis in plasma, exerted no pharmacological activity (Table 2). It is worth mentioning that there have been reports of aminoethyl esters of naproxen and ketorolac, but they have not been evaluated for possible anticholinergic activity [7,80].

### 3.3. AChEI-NSAIDs

**Figure 19:**
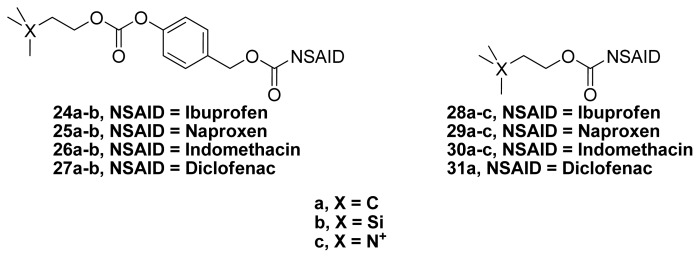
The chemical structures of AChEI-NSAIDs **24a**–**b**, **25a**–**b**, **26a**–**b**, **27a**–**b**, **28a**–**c**, **29a**–**c**, **30a**–**c** and **31a**.

Young *et al.* have reported the synthesis and evaluation of the NSAID ester–carbonates **24a**–**b**, **25a**–**b**, **26a**–**b** and **27a**–**b** and the NSAID esters **28a**–**c**, **29a**–**c** and **30a**–**c**, in addition to the diclofenac ester **31a**, as AChEI-NSAIDs (Figure 19). These compounds contain choline (**c**, **X** = **N^+^**) or its carbon (**a**, **X** = **C**) and silicon (**b**, **X** = **Si**) bioisosteres.

The authors have demonstrated that these derivatives exhibit an acetylcholinesterase inhibitory activity and, hence, called them AChEI-NSAIDs [81,82]. Table 3 shows the profile of some of the compounds that have been fully tested by those authors. The choline-like quaternary nitrogen, or its carbon and silicone bioisosteres, is essential for activity. These drugs are designed to reduce the inflammatory response caused by sulfur mustard (SM, 2,2′-dichloroethyl sulfide). As seen in Table 3, these agents were effective as AChEIs, although less potent than tacrine, and were able to reduce the inflammatory response more effectively than their parent NSAIDs. The potency of these compounds is related to AChEI activity, lipophilicity and longer half-lives [82]. It is likely that the observed anti-inflammatory effect is due to a combination of the intrinsic AChEI activity of the intact molecule and the anti-inflammatory effect of the liberated NSAID, as the hydrolysis half-lives of such compounds ranges from less than 5 min up to 468 min [81].

To conclude, the anticholinergic NSAIDs and the AChEI-NSAIDs are designed to have dual activity, one for the whole intact molecule and another for the parent NSAID. These NSAID esters do not fit the definition of codrug (mutual prodrug), because the connected promoiety (*i.e.*, the aminoethyl moiety that is connected to the NSAID to form the prodrug) is not pharmacologically active on its own, nor do they fit the strict definition of prodrugs.

## 4. Phospho-NSAIDs

### 4.1. Introduction

**Figure 20:**
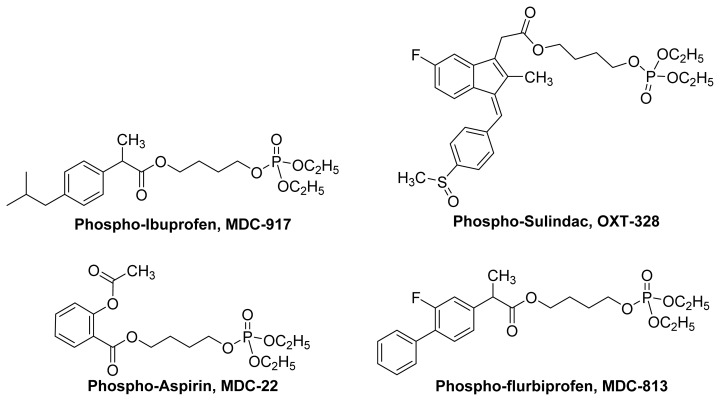
The chemical structures of some Phospho-NSAIDs.

Phospho-NSAIDs are intriguing compounds that consist of an NSAID molecule that is connected to dialkylphosphate via a linker (Figure 20). Structurally, phospho-NSAIDs can be regarded as diethylphosphate analogs of nitrate NO-NSAIDs. This group of compounds has been extensively tested, especially phospho-aspirins, as anticancer agents and, to a lesser extent, as anti-arthritis drugs. Actually, NSAIDs themselves exhibit antineoplastic activity, but their less than optimum efficacy precludes their use as anticancer agents [83].

### 4.2. Mechanism of Action Phospho-NSAIDs

Phospho-sulindac (**PS**, **OXT-328**), phospho-ibuprofen (**PI**, **MDC-917**) and phospho-aspirin (**PA**, **MDC-22**) have been evaluated as anti-arthritis agents and were found to be effective in relieving joint inflammation and edema, with reduced GI toxicity. It is interesting to note that **OXT-328** and **MDC-917** can suppress the synthesis of PGE_2_*in vitro*, whereas **MDC-22** only inhibits its production *in vivo*, like a true prodrug [84].

**Figure 21:**
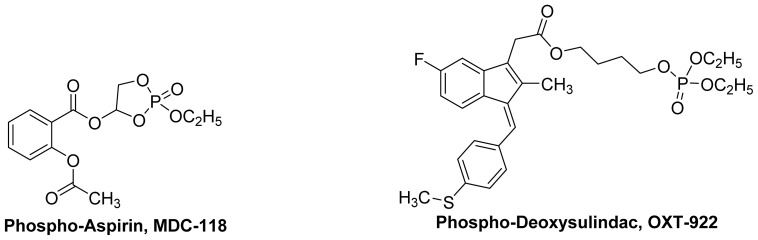
The chemical structures of phospho-aspirin (**MDC-118**) and phospho-deoxysulindac (**MDC-922**).

With regard to anticancer activity, **OXT-328**, **MDC-917 and MDC-813** (Figure 20) and **MDC-118** and **MDC-922** (Figure 21) have been shown to inhibit tumor growth by suppressing cell proliferation and enhancing apoptosis. **OXT-328** and **MDC-118** were shown to be effective *in vivo* with no detectable animal toxicity [83].

Phospho-NSAIDs are proposed to act through a COX-independent mechanism, such as the inhibition of the thioredoxin system and a redox sensitive transcription factor NF-κB [83,85] and/or induction of RONS (reactive oxygen and nitrogen species) [85]. Phospho-deoxysulindac (**OXT-922**) inhibits the growth of human colon cancer cell via a redox/polyamine-dependent mechanism of action [86]. **MDC-917** is also effective against colon cancer [87].

*In vivo*, it was found that *carboxylesterases 1* and *2* can hydrolyze phospho-NSAIDs. In this regard, *carboxylesterase-1* hydrolyzes phospho-sulindacs, phospho-ibuprofens, phospho-naproxens and phospho-indomethacins preferentially, whereas *carboxylesterase-2* preferentially targets phospho-aspirins. Recently, it has been determined that the intact phospho-NSAID molecule is required for its anticancer activity, and that inhibition of *carboxylesterases* enhances the efficacy of phospho-NSAIDs both *in vitro* and *in vivo*[88].

**Figure 22:**
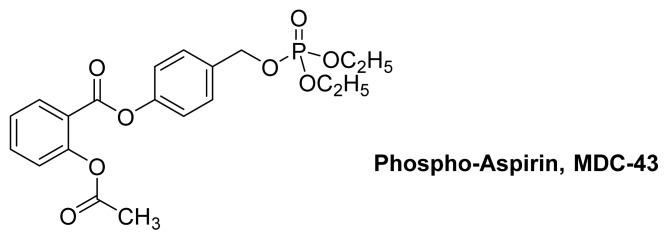
The chemical structure of phospho-aspirin **MDC-43**.

The phospho-aspirin **MDC-43** (Figure 22) is reported to be effective against human cancer cell lines [89]. A study of the SAR of **MDC-43** and its analogs revealed that the aromatic ring is important for inducing apoptosis. The proposed mechanism of action is similar to that proposed for the NO-NSAID **NCX-4040** (Figure 14), which shows aspirin as the promoiety and not the parent drug, and here, the phosphate group plays the role of the leaving group [64]. In this context, **MDC-43** and **NCX-4040** are regarded as prodrugs for *p*-quinone methide [90].

To conclude, it seems that phospho-NSAIDs may have anticancer and cancer chemopreventive activity as potential and important alternative pharmacological actions for classical NSAIDs.

## 5. Miscellaneous Agents

### 5.1. TEMPO-NSAIDs

**Figure 23:**
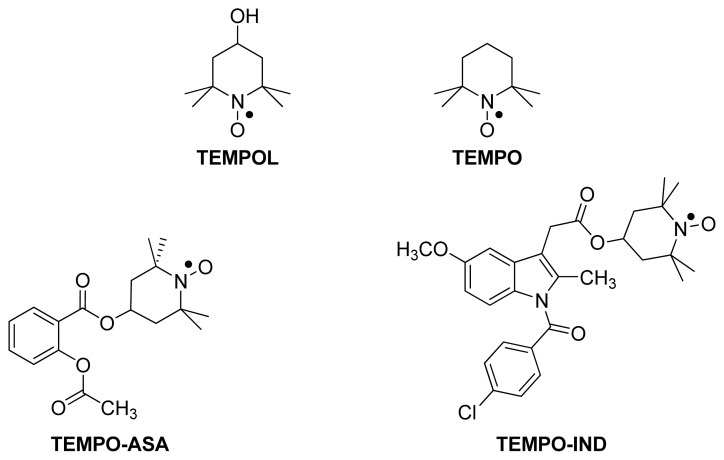
The chemical structures of the TEMPOL esters of aspirin (**TEMPO-ASA**) and of indomethacin (**TEMPO-IND**).

2,2,6,6-Tetramethyl-1-piperidinyloxy (**TEMPO**) and 4-hydroxy-TEMPO (**TEMPOL**) (Figure 23) are chemically stable nitroxides (nitroxyl radicals) that can play an antioxidant role through radical-radical or radical-scavenging reactions with Reactive Oxygen Species (ROS). Pharmacologically, TEMPOL has been shown to prevent the aggravation of indomethacin-induced lesions in the stomach [91]. Flores-Santana *et al.* (2010) have reported the synthesis and the pharmacological evaluation of the TEMPOL ester of aspirin (**TEMPO-ASA**) and indomethacin (**TEMPO-IND**) [92], Figure 23.

The two TEMPO-NSAIDs were able to scavenge superoxide via the TEMPO parts of the esters. In addition, the two compounds inhibited the production of PGE_2_*in vitro* with an IC_50_ of 30 μg/mL for **TEMPO-ASA** and 10 μg/mL for **TEMPO-IND**, values comparable to those of their parent NSAIDs. What was interesting is that the esters also inhibited the production of Leukotriene B_4_ (LTB_4_) *in vitro*, whereas aspirin, indomethacin and TEMPOL were inactive. This is a new pharmacological property that can only be attributed to the intact form of these esters. LTB_4_ is a very potent activator of leukocytes and plays a major role in chemotaxis. It is involved in various inflammatory processes, and there are LTB_4_ receptor antagonists that are used as anti-inflammatory drugs [93]. With regard to safety, the maximum tolerable dose (MTD) of **TEMPO-ASA** was not higher than that of aspirin, whereas the MTD of **TEMPO-IND** was increased by eight-fold. This finding might indicate that the *in vivo* hydrolysis of **TEMPO-ASA** is faster than that of **TEMPO-IND**, hinting at the possibility that the intact molecule is responsible for the observed safety profile. **TEMPO-IND** was tested further for *in vivo* anti-inflammatory activity and ulcerogenicity index and was found to be 15% more active than indomethacin and about 10 times less ulcerogenic [82].

### 5.2. HS-NSAIDs

Physiologically, hydrogen sulfide (H_2_S) has been implicated in both pro-inflammatory and anti-inflammatory responses, depending on the organ and/or dose [94].

**Figure 24:**
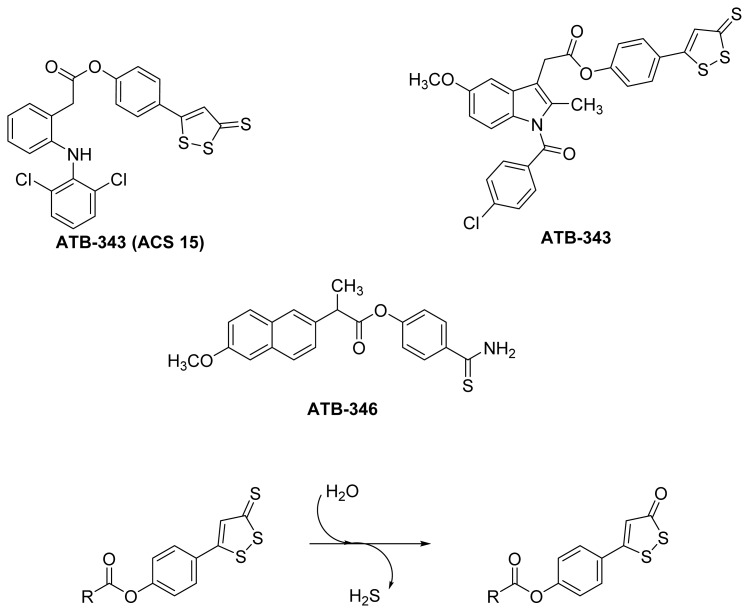
The chemical structures of some H_2_S-releasing NSAIDs (HS-NSAIDs).

It has been reported that gastric mucosal injury caused by NSAIDs is reduced by H_2_S without reducing the NSAIDs’ effect on prostaglandin biosynthesis [95]. Actually, it was shown that H_2_S donors, such as 4-hydroxythiobenzamide (4HTB), can induce better ulcer healing [96]. These findings have prompted the design and synthesis of hydrogen sulfide-releasing NSAIDs (HS-NSAIDs). Examples and the mechanism of H_2_S release [94] from the most commonly used H_2_S donating moiety, dithiolethione, are presented in Figure 24.

Although a detailed mechanism for the release of hydrogen sulfide from dithiolethiones was not proposed, it has been demonstrated that dithiolethione derivatives gradually disappear with the concurrent appearance of the corresponding 1,2-dithiole-3-one upon heating to 120 °C in DMSO-aqueous phosphate buffer system (100 mM, pH 7.4), implying hydrolysis as the possible mechanism of hydrogen sulfide liberation [97].

**Figure 25:**
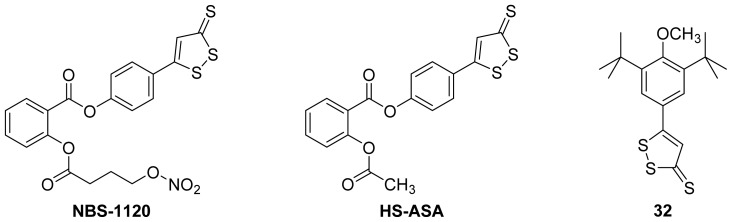
The chemical structures of **NOSH-ASA**, **HS-ASA** and the dithiolethione derivative **32**.

An interesting example is the salicylic acid ester **NBS-1120** (Figure 25), which was actually designed as an H_2_S- and NO-releasing NSAID and, hence, was dubbed a NOSH-NSAID. It has been demonstrated that the effect of H_2_S donors are significantly augmented when combined with nitroglycerin, an NO donor [96]. Although **NBS-1120** has anti-inflammatory activity comparable to aspirin, it has an IC_50_ of 48 nM against colon cancer cells, an action that is thought to be mediated by a COX-independent mechanism [98]. In contrast, **SH-ASA** (Figure 25), was much less active when tested against the same colon cancer cell line (HT-29) with an IC_50_ of only 48 μM [99]. Further studies showed that in addition to the exhibited *in vitro* and *in vivo* anticancer activity of **NBS-1120**, it caused the induction of apoptosis and inhibition of cell proliferation [100]. Furthermore, it was clearly shown that **NBS-1120** was 1000 times more potent in HT-29 cells than a combination of aspirin, a H_2_S donor, and a NO donor, which is strong evidence that the intact molecule is required for the observed potent anticancer activity [100]. The *in vivo* anti-inflammatory profile of **NBS-1120** was similar to that of aspirin, with greater inhibition of COX-1, implying that, in this regard, it is a true salicylic acid prodrug. Finally, it is worth mentioning that the hydrogen sulfide donor **32** (Figure 25) was more potent than rofecoxib as a COX-2 inhibitor with a 500-fold COX-2/COX-1 selectivity [97].

## 6. Conclusions

Any newly synthesized chemical entity might or might not have intrinsic pharmacological activity, and that holds true for compounds that are synthesized as potential prodrugs. NSAID prodrugs represent an effective approach to achieve the desired anti-inflammatory action with a significant reduction of gastric side effects. In general, the reduction in these side effects is mainly attributed to the masking of the carboxylic acid group. NO-NSAIDs were designed to add another dimension to the GIT safety profiles of NSAID prodrugs, namely the protective action of NO released *in vivo*. Furthermore, it would be reasonable to speculate that NO-NSAIDs that possess intrinsic selective COX-2 inhibitory action might be formidable alternatives to current selective COX-2 inhibitors, as nitric oxide has a vasodialtory action that can reduce the cardiovascular complications observed with Coxibs. In addition, purposeful design of NSAID prodrugs that have anticholinergic action may offer another approach to GIT safety, with AChEI-NSAIDs leading to augmented anti-inflammatory action. The intriguing case of phospho-NSAIDs, which seems the most complicated of all, may allow for the utilization of these otherwise OTC medications in the fight against cancer. It is clearly evident that the mechanism of action of “NSAID prodrugs” discussed in this review is more complicated than simply masking the carboxylic acid, releasing NO or H_2_S or COX-related anticancer activity. It is really difficult to give a specific designation to “prodrugs” with intrinsic pharmacological activity. The term *Hybrid Prodrugs* might seem appealing, but it is not well defined. For the time being, the compounds discussed in this review are diverse and cannot be classified under one umbrella.

## Figures and Tables

**Table 1:** Some nitric oxide-releasing moieties and examples of NO-NSAIDs that contain them.

Name	NO donor moiety	Equivalents of NO released	Examples
Nitrates	–ONO_2_	1	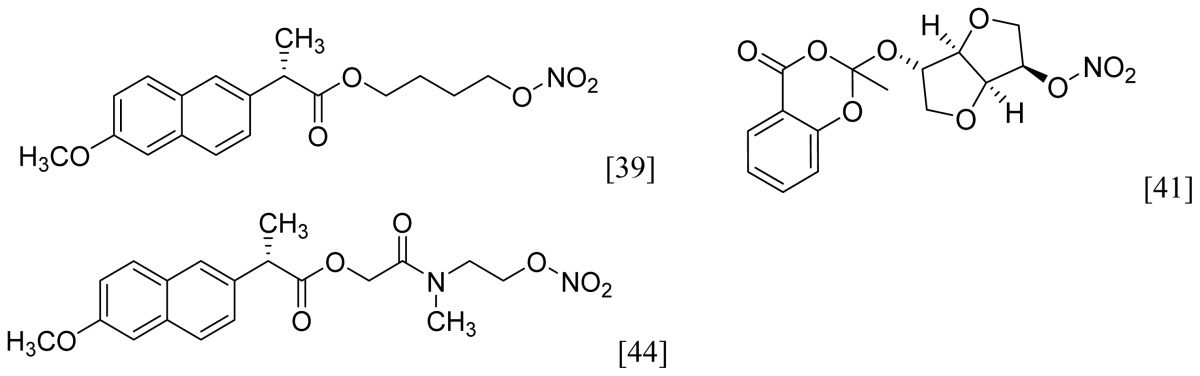
NONOate	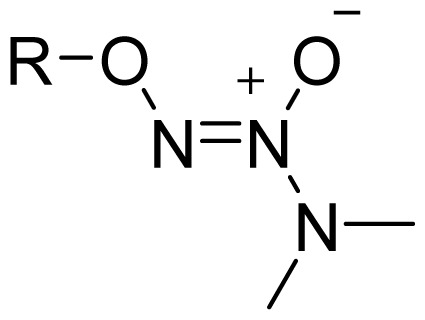	2	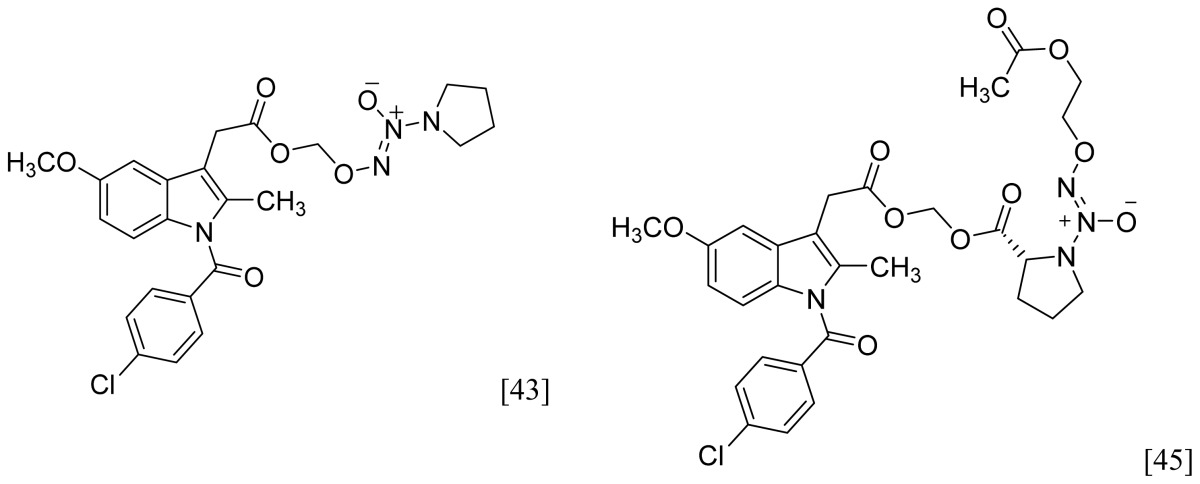
Furoxan and Benzofuroxan	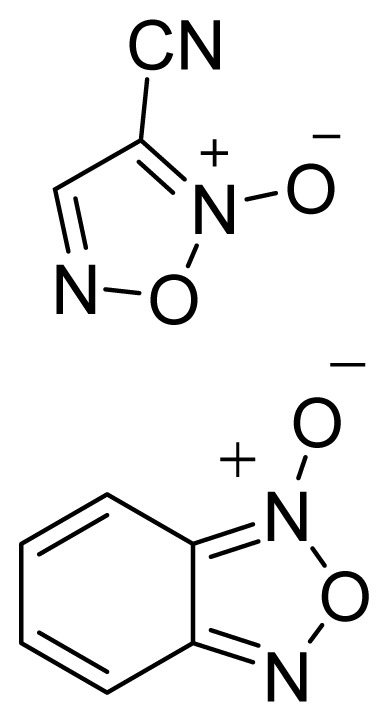	1	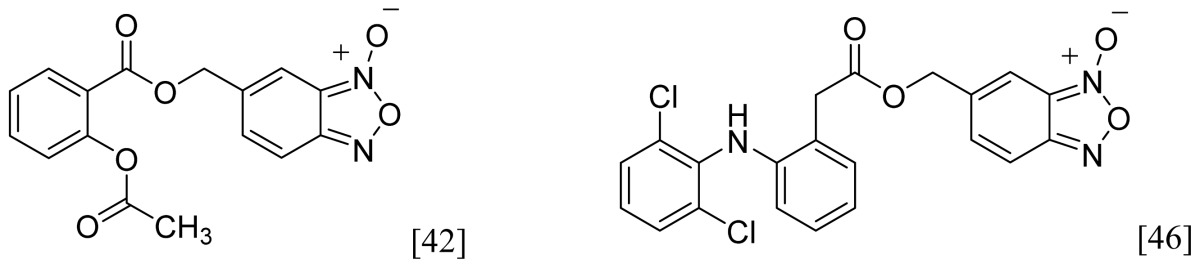
Sulfohydroxamic Acid	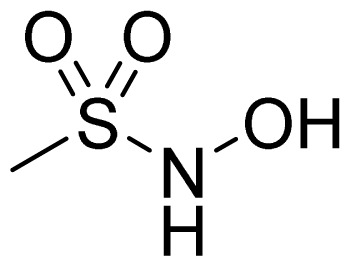	1	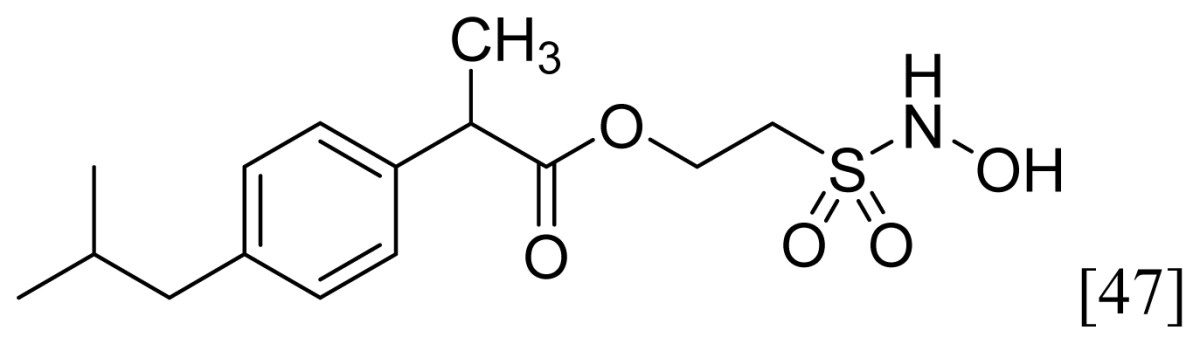
Nitrosothiol Esters	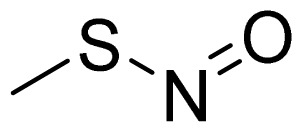	1	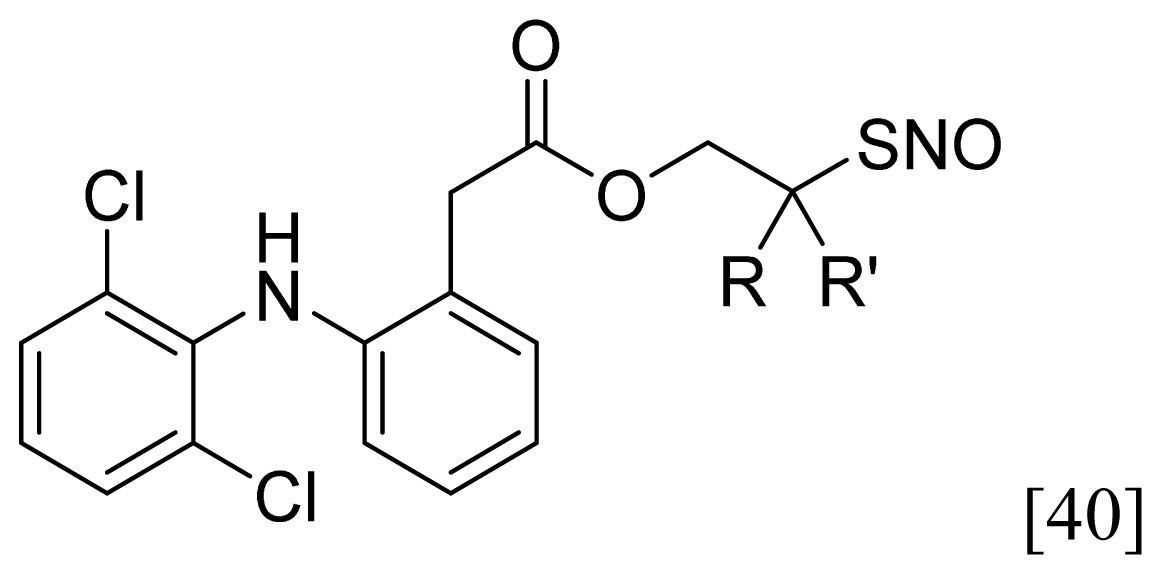

**Table 2:** The plasma half-life (*t*_1/2_), % parent NSAID released, the *in vivo* biological activity (pA_2_ and % inhibition of edema volume,) and the Ulcerogenic Index (UI) of the aminoethyl esters of ketoprofen **21a**–**f**, flurbiprofen **22a**–**22f** and indomethacin **23a**–**e**.

Compound	*t*_1/2_, pH 7.4 (h)	% NSAID released in plasma after 2 h	pA_2_**	% inhibition of Edema Volume	UI
Ketoprofen a				87	0.882
21a–e a	16–64	58.9–79.6	5.28–6.43	70–89	0.306–0.376
21f a	ND *	ND *	7.58	86	0.299
Flurbiprofen b				0	0.800
22a–e b	121–505	17.9–51.1	4.73–5.79	79–89	0.130–0.230
22f b	ND *	ND *	6.31	0	0.00
Indomethacin c				45	0.55
23a–e c	34–99	49.6–79.9	4.49–5.19	17–46	0.130–0.320
Atropine a,b,c			8.02		

* ND: No detectable hydrolysis was observed;

** pA_2_: Anticholinergic activity;

a For anti-inflammatory activity, a 20 mg/kg dose was used, and for UI, a 200 mg/Kg dose was used [74];

b For anti-inflammatory activity, a 0.03 mmol/kg dose was, and for UI, a 0.93 mmol/Kg dose was used [71];

c For anti-inflammatory activity, a 3 mg/kg dose was used, and for UI, a 25 mg/Kg dose was used [73].

**Table 3:** The *in vivo* biological activity of some AChEI-NSAIDs (% reduction in edema and acetylcholinesterase inhibition) and their plasma *t*_1/2_ and calculated LogP.

Comd	% reduction in edema (Mouse ear vesicant model) [81]		Acetylcholinesterase Inhibition [72]	Plasma *t*_1/2_ (min)	Calculated LogP
			
	CEES	TPA	IC_50_ (μM)		
24a	20	41	1.93 ± 0.64	204	7.37
25b	45	70	0.83 ± 0.15	253	6.67
26a	91	21	2.29 ± 0.94	468	7.87
27a	90	24	0.51 ± 0.02	357	8.41
31a	113	29	2.69 ± 0.15	111	7.52
Tacrine	-	-	0.055 ± 0.005	-	-
DIC	17	58	-	-	-
IND	46	55	-	-	-
IBU	-15	-33	-	-	-
NAP	NS	*	104	-	-

CEES: 2-Chloro-ethyl-ethyl sulfide. TPA: 12-*O*-Tetradecanoylphorbol-13-acetate. NS: No significant reduction.
